# Supplementary material for: Highly conductive tissue-like hydrogel interface through template-directed assembly
Source: Nat Commun. 2023 Apr 18;14:2206. doi: 10.1038/s41467-023-37948-1 (PMC10113367; doi:10.1038/s41467-023-37948-1)
Supplement: Supplementary file 1 — Supplementary Information [file 41467_2023_37948_MOESM1_ESM.pdf]

Supplementary information

Highly conductive tissue-like hydrogel interface through template-directed assembly

Jooyeun Chong,[1†] Changhoon Sung,[2†] Kum Seok Nam,[2] Taewon Kang,[1] Hyunjun Kim,[1] Haeseung Lee,[1] Hyunchang Park,[1] Seongjun Park,[2,3]* and Jiheong Kang[1,3]*

[1] Department of Materials Science and Engineering, Korea Advanced Institute of Science and Technology (KAIST), Daejeon, 34141 Republic of Korea

[2] Department of Bio and Brain Engineering, Korea Advanced Institute of Science and Technology (KAIST), Daejeon, 34141 Republic of Korea

[3] KAIST Institute for NanoCentury, Daejeon, 34141 Republic of Korea

[†]These authors contributed equally: Jooyeun Chong, Changhoon Sung

* To whom correspondence should be addressed:

E-mail: spark19@kaist.ac.kr (S.P.), jiheongkang@kaist.ac.kr (J. K.)

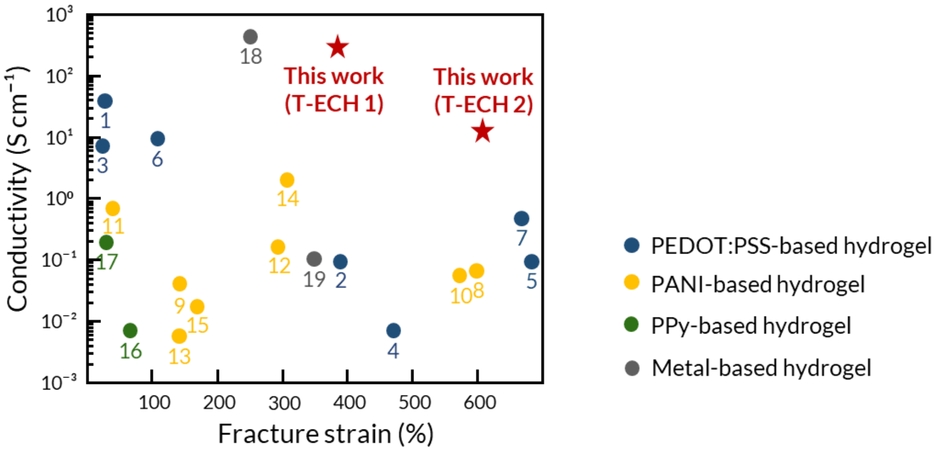

Supplementary Fig. 1| Ashby plot showing fracture strain and conductivity of conductive hydrogels. Supplementary references 1-19.

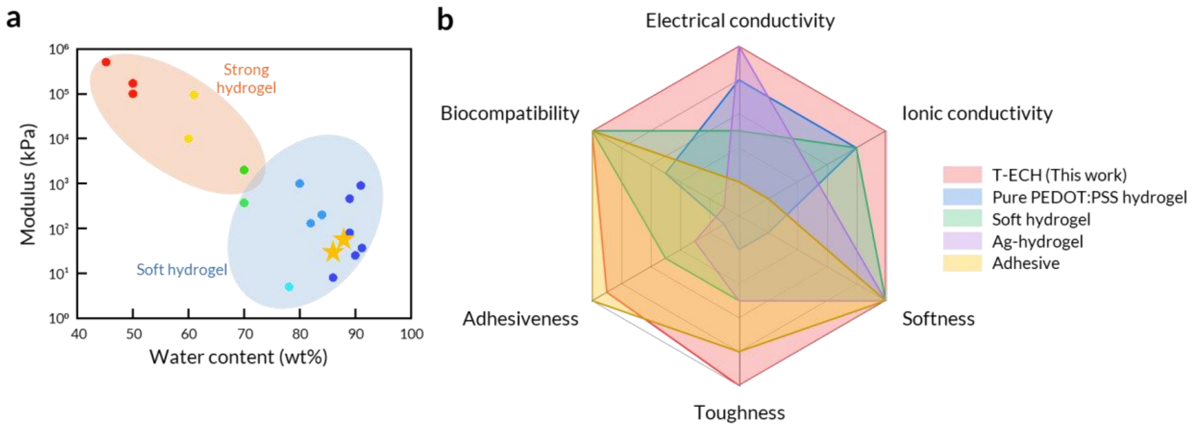

Supplementary Fig. 2| Comparison of T-ECH and other hydrogels.

a, Ashby plot showing water content and elastic modulus of hydrogels[6, 18, 20-34]. **b**, A comprehensive comparison between T-ECH of this work and previously reported conductive hydrogels in terms of electrical conductivity, ionic conductivity, softness, toughness, adhesiveness, and biocompatibility including pure PEDOT:PSS hydrogel[1], soft hydrogel[20], Ag-hydrogel[18], and adhesive[35].

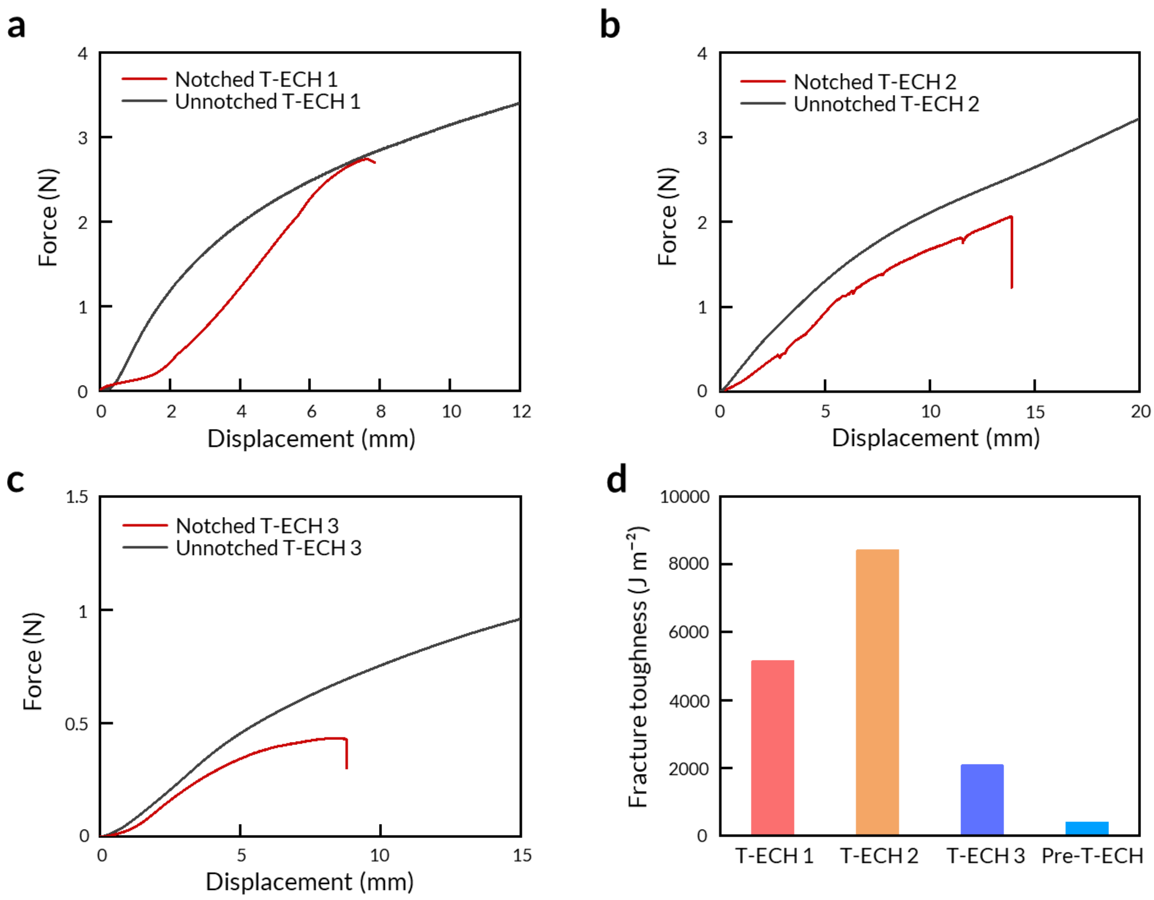

Supplementary Fig. 3| Fracture toughness of T-ECHs.

a, **b**, **c**, Force vs. displacement curves of T-ECHs with (red) and without (black) a notch. **d**, Fracture toughness of T-ECHs and Pre-T-ECH.

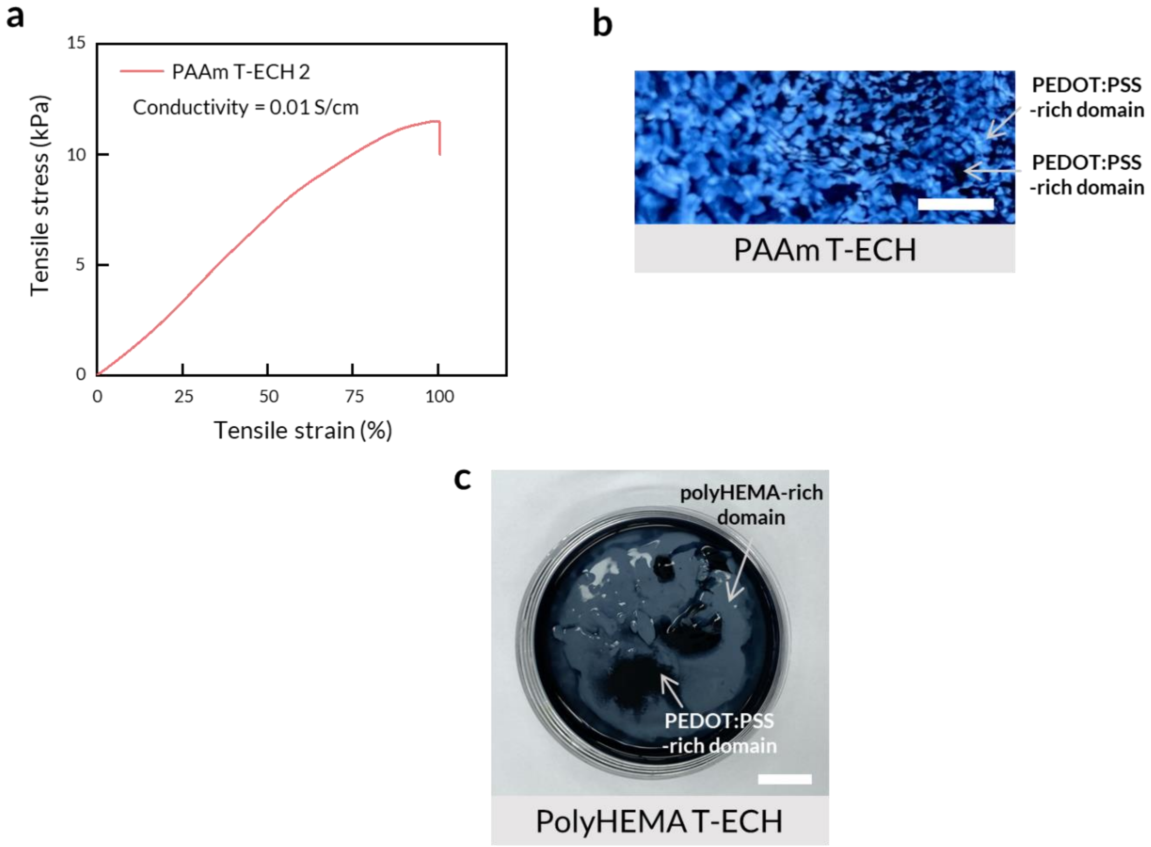

Supplementary Fig. 4| T-ECH using PAAm and PolyHEMA as the template polymer.

a, Stress-strain curve of PAAm T-ECH 2, with 0.5 mol% of crosslinker and 5 wt% of PEDOT:PSS. The electrical conductivity of PAAm T-ECH 2 is 0.01 S/cm. **b**, Image of PAAm T-ECH taken under the flashlight. PAAm T-ECH shows phase-separated domains of PEDOT:PSS and PAAm (Bright area: PAAm, dark area: PEDOT:PSS). Scale bar = 3 mm. **c**, Image of poly(2-hydroxyethyl methacrylate) (polyHEMA) T-ECH. It was not able to perform mechanical tests because hydrogel could not be synthesized using HEMA and PEDOT:PSS. This phenomena comes from the high energic disagreement between PEDOT:PSS and polyHEMA due to the low hydrogen interaction between PEDOT:PSS and polyHEMA. It shows significant phase separation of PEDOT:PSS and polyHEMA. Scale bar = 1 cm.

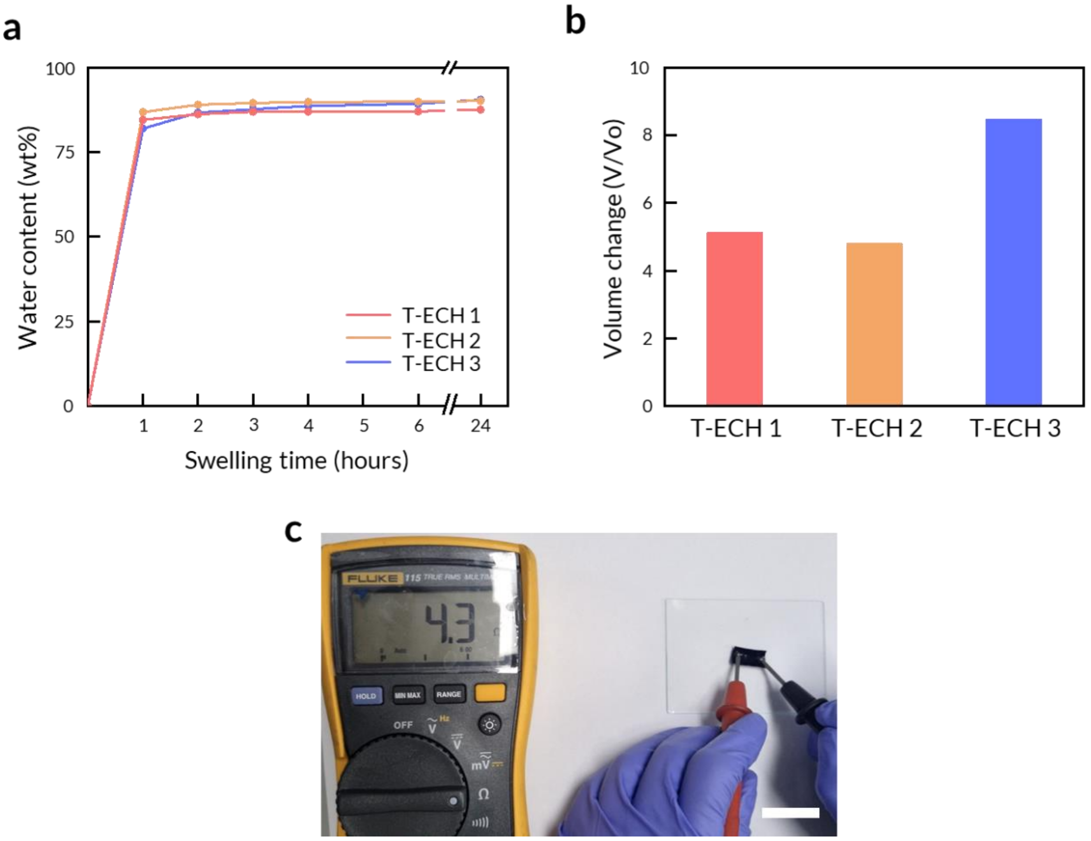

Supplementary Fig. 5| Swelling behaviors of T-ECHs in water.

a, Weight change of T-ECHs during swelling in water. **b**, Volume change of T-ECHs in water for 24 h. V_o is the volume of T-ECH before swelling in water. **c**, Image of measuring the resistance of T-ECH 1 with a multimeter (resistance = 4.3 Ω). Scale bar = 2 cm.

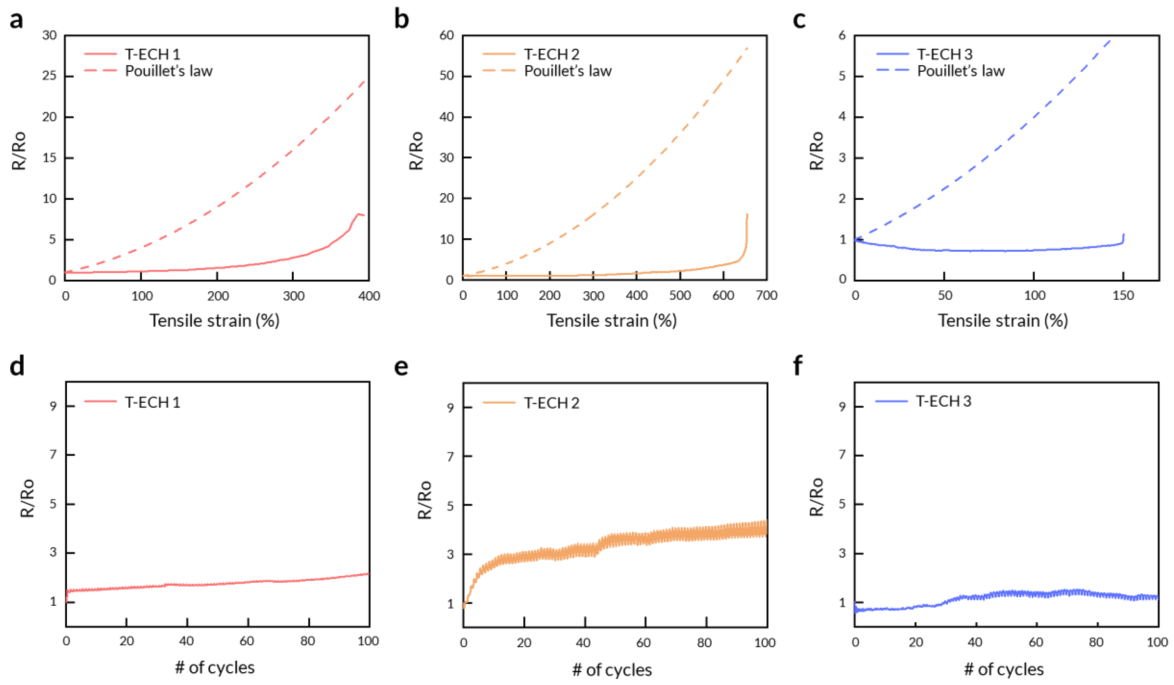

Supplementary Fig. 6| Resistance change during stretching and cyclic stretching.

a, **b**, **c**, Resistance change during the strain of T-ECHs. Strain rate = 200%/min. **d**, **e**, **f**, Resistance change during the cyclic strain of 100%. Strain rate = 300%/min. (R_0 = 5 Ω (T-ECH 1), 108 Ω (T-ECH 2), 82 Ω (T-ECH 3).

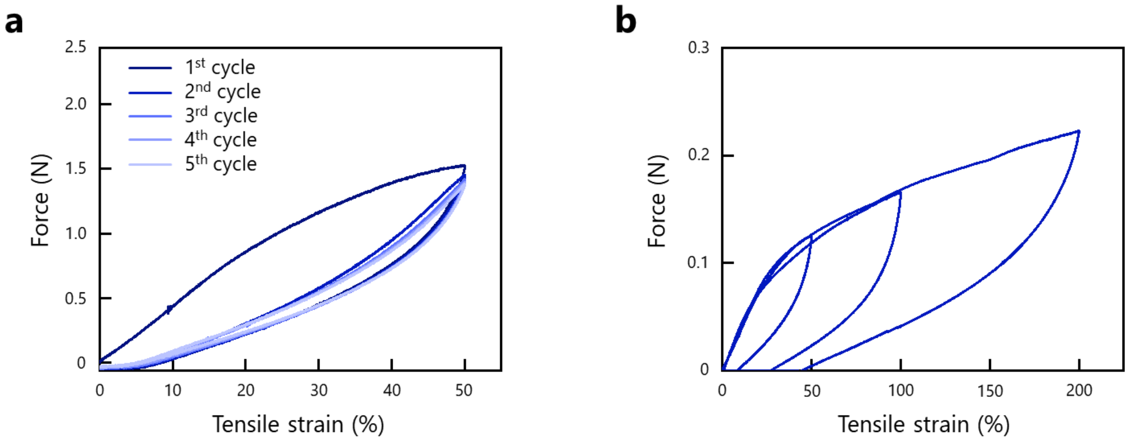

Supplementary Fig. 7| Cyclic tensile test of T-ECH.

a, Cyclic tensile test was done for 5 cycles with 50% strain and a strain rate of 100%/min. **b,** Cyclic tensile test with different strains (50%, 100%, and 200%) with a strain rate of 100%/min.

When stretched to 50%, T-ECH showed a low residual strain of 5%. Also, when the tensile strain is 200%, the residual strain is less than 50%, meaning that it has reversible stretchability. Although T-ECH has a double-network structure of PEDOT:PSS and PAA, the composition of PEDOT:PSS is much lower than PAA. Accordingly, the mechanical destruction while stretching is low due to the stretchable and reversible nature of PAA. Therefore, it can have low residual strain and can be used for practical applications.

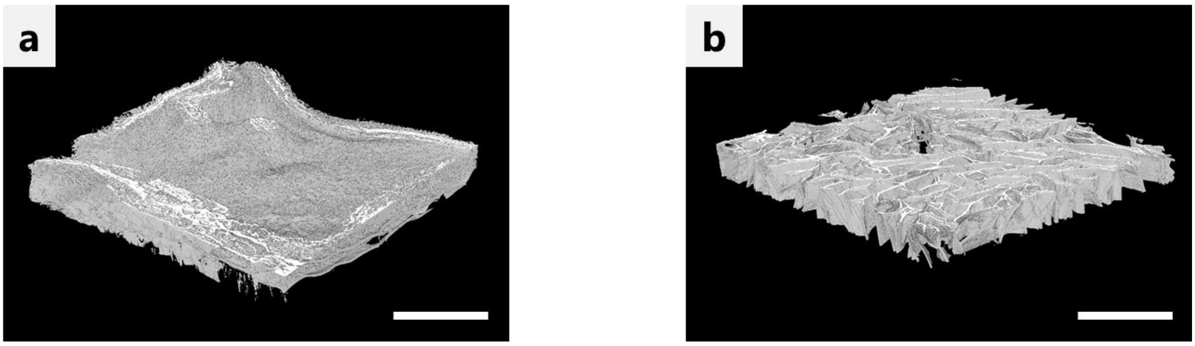

Supplementary Fig. 8| Micro-CT images.

Micro-CT images of T-ECH (**a**) and Pure ECH (**b**). T-ECH has a micro-porous network of hydrogel while Pure ECH shows huge phase separation of brittle, crystalline domains. Scale bar = 1 mm.

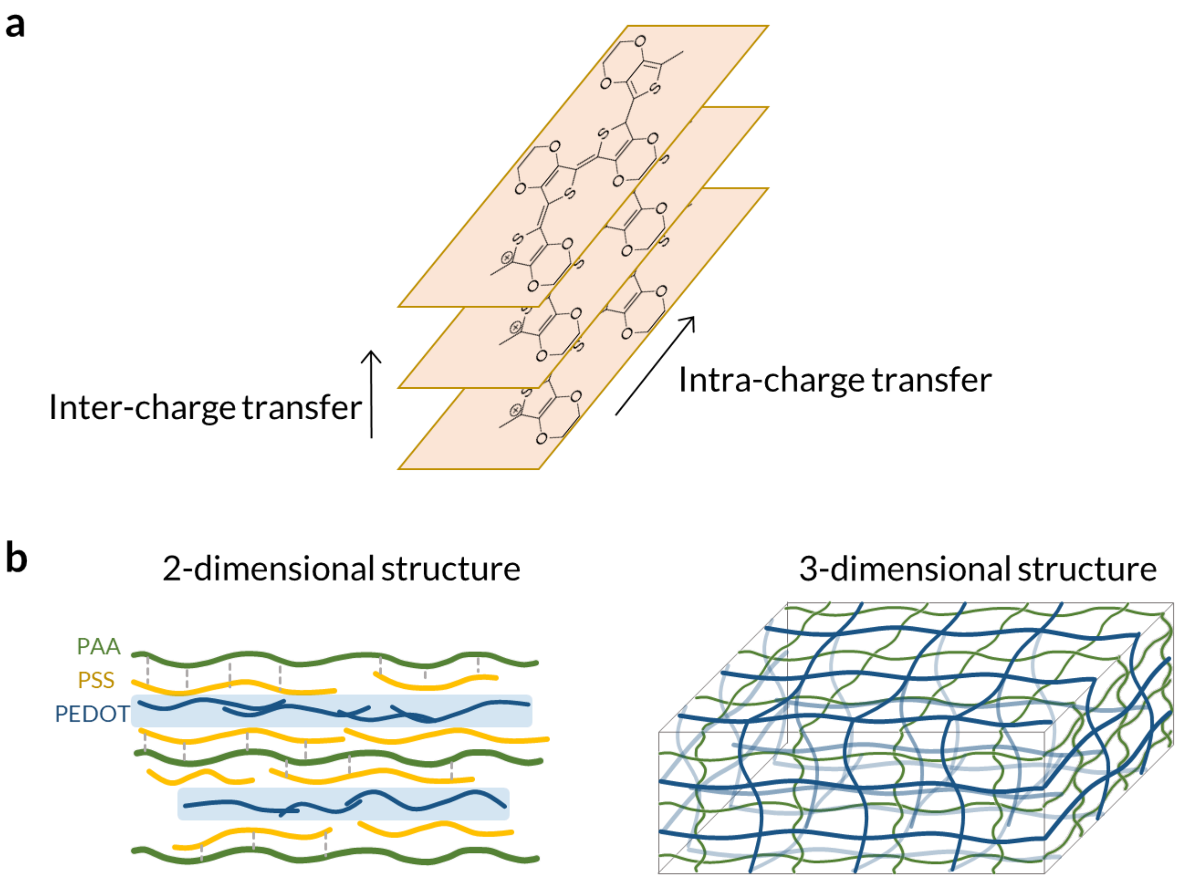

Supplementary Fig. 9| Electrically conductive paths made in T-ECH.

a, Schematic illustrating of inter-, intra-charge transfer in the conductive polymer. **b**, Schematic illustrating of intra-transfer pathways made in linearly connected PEDOT networks in T-ECH along the template PAA network. Because PEDOT in T-ECH is unentangled and has a linear conformation, the intra-charge transfer is facilitated[36, 37]. The unentangled PEDOT chains have a high degree of intrachain and interchain ordering, which facilitates electrical conduction. The conductive pathways are well-made to have high electrical and ionic conductivities.

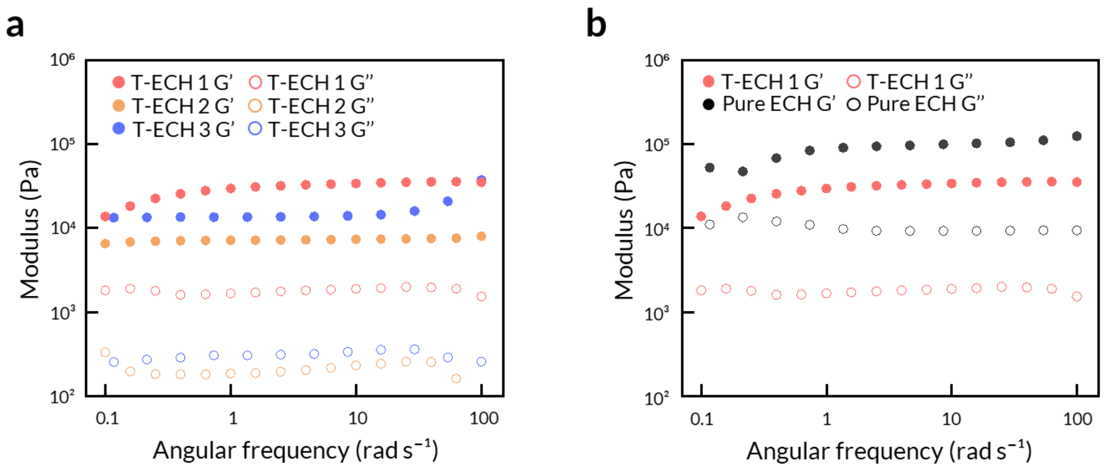

Supplementary Fig. 10| Rheological characteristics of T-ECHs and Pure ECH.

a, Frequency sweep measurements of T-ECHs. All T-ECHs show stable hydrogel behaviors. **b**, Frequency sweep measurements of T-ECH 1 and Pure ECH. The storage modulus of T-ECH is lower than Pure ECH, implying that T-ECH has less entangled network than Pure ECH which has only highly entangled PEDOT:PSS aggregates. Therefore, electrical conduction in T-ECH is faster than Pure ECH due to the high intra- and inter-chain ordering.

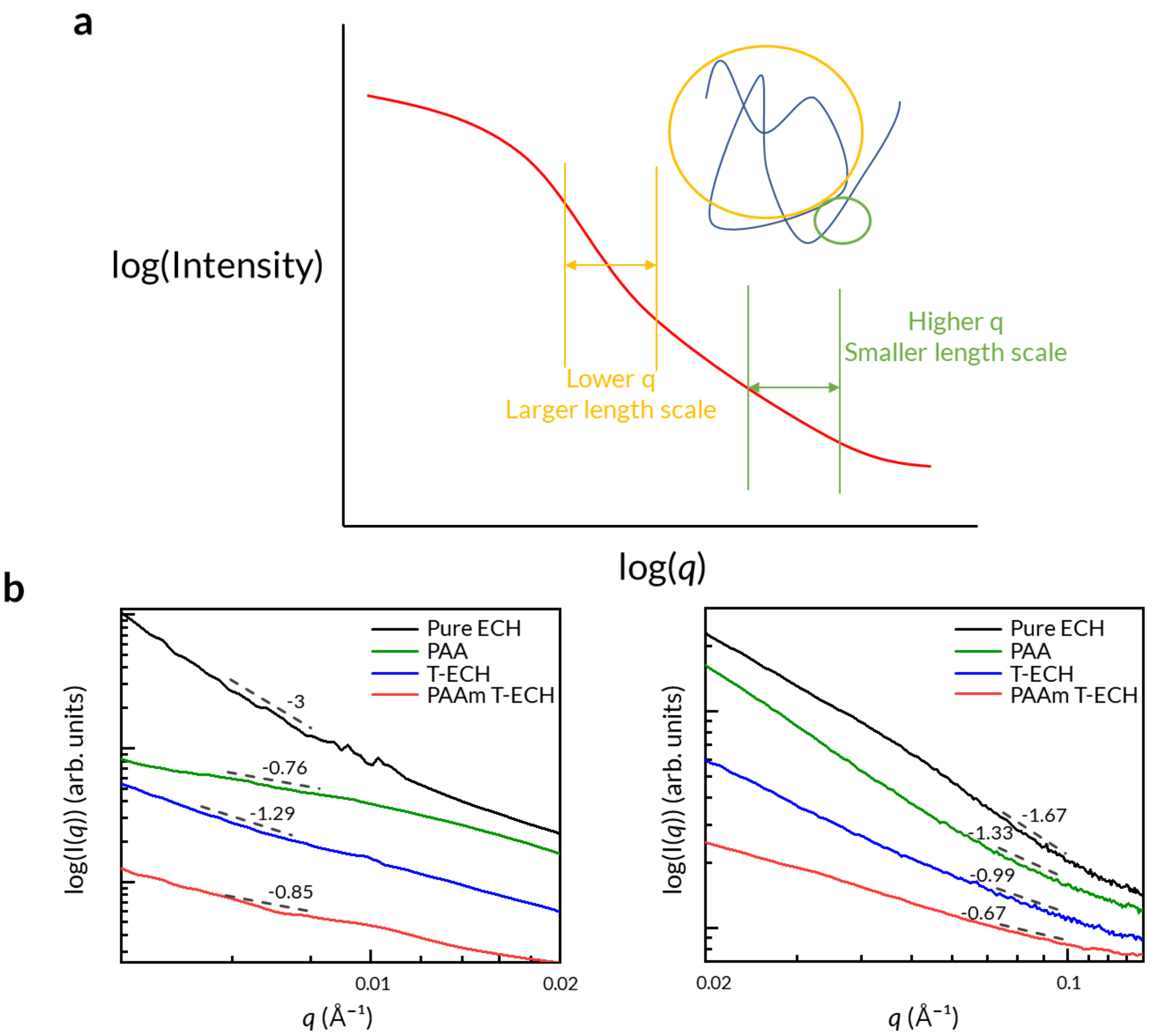

Supplementary Fig. 11| SANS analysis.

a, Structural information of the gel structure can be obtained from SANS measurement. For q > 1/Rg (radius of gyration), morphological information of polymer chains is revealed via power-law decay, I ~ q^{-n}. The exponent, n, is directly related to the particle geometry. **b**, The values of Rg of hydrogels were all determined to be less than 400 Å according to the Guinier-porod model (Supplementary Table 2). Small q ranges (1/Rg < q < 0.01 $Å^{-1}$) correspond to a large morphological measuring scale (> 628 Å). The n value of Pure ECH is 3, meaning that thick rod-like PEDOT:PSS fibers are formed at this length scale (> 628 Å). In Pure ECH, the bulk aggregates of PEDOT and PSS led to large bulky fibers, greater than 600 Å. On the other hand, T-ECH has an n value of 1.29, indicating that it has a structure of a gaussian polymer chain or needle. Therefore, we can conclude that PEDOT in T-ECH has a linear and thin fibrous network, contrary to Pure ECH. However, in PAAm T-ECH, since the template polymer cannot

induce a PEDOT network, it shows no network formation of PEDOT similar to pure PAA hydrogel.

For mid-range angles (0.03 $Å^{-1} < q < 0.1$ $Å^{-1}$), corresponding to a smaller distance (60-200 Å), we can evaluate how complicated and compact the structure is from the exponent, n. Pure ECH, with an n value of 1.67, shows the most compact structure having highly entangled PEDOT and PSS fibers. Because T-ECH has a rigid, linear, and extended chain structure of PEDOT, its n value of 0.99 is lower than that of PAA, 1.33, which has only soft and flexible polymer chains. The linear and rigid PEDOT network leads to a less complicated structure.

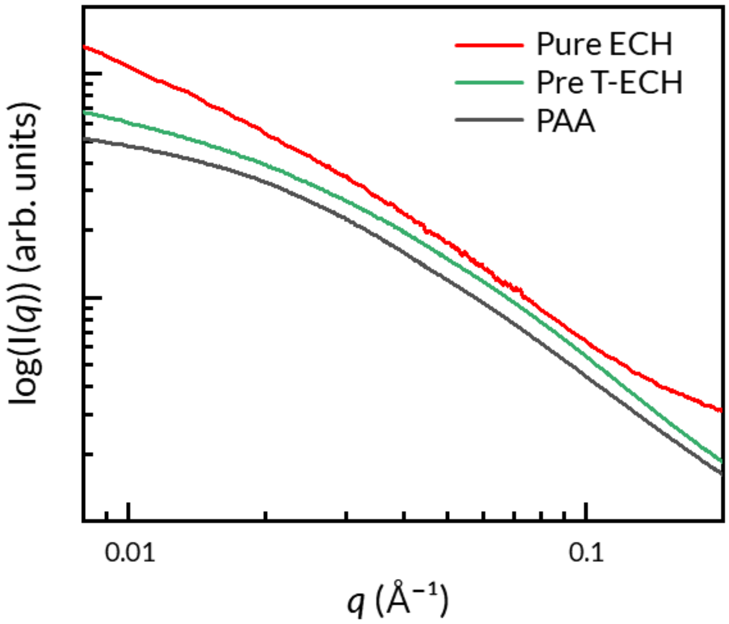

Supplementary Fig. 12| SAXS analysis of Pre T-ECH and T-ECH.

Pre T-ECH and PAA have similar features, meaning that there is no significant conformational difference between Pre T-ECH and PAA. This result explains that colloidal PEDOT:PSS in Pre T-ECH doesn't make a fibrous network. However, T-ECH showed PEDOT:PSS fibrous network structure inside the PAA template network. Specifically, T-ECH showed a higher slope at a low *q* range ($q < 0.01$ Å-1) than Pre T-ECH and PAA, indicating that a needle-like PEDOT network is made inside PAA. At a mid *q* range ($q > 0.1$ Å-1), T-ECH had a lower slope than Pre T-ECH and PAA due to the linearity of PEDOT chains made in complex and flexible PAA chains. Therefore, through DMSO annealing and re-swelling, PEDOT:PSS colloids in Pre T-ECH transform into the fibrous network in T-ECH.

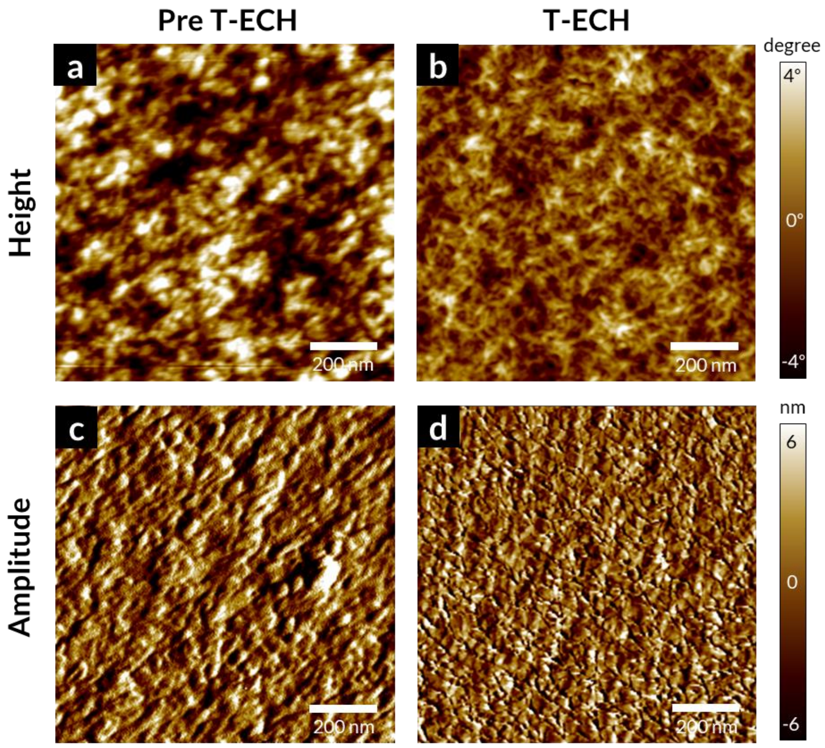

Supplementary Fig. 13| AFM height and amplitude images of Pre T-ECH and T-ECH.

a, **b**, Height image of Pre T-ECH, T-ECH. **c**, **d**, Amplitude image of Pre T-ECH, T-ECH. From the AFM image, conformational change from Pre T-ECH to T-ECH was observed. The DMSO annealing process transformed colloidal PEDOT:PSS to thin fibrous PEDOT:PSS network, making ordered PEDOT:PSS/PAA ordered structure. T-ECH has a nanofibrous network of PEDOT:PSS whereas no such fibrous network is seen in Pre T-ECH. Similar results were observed in two independent samples.

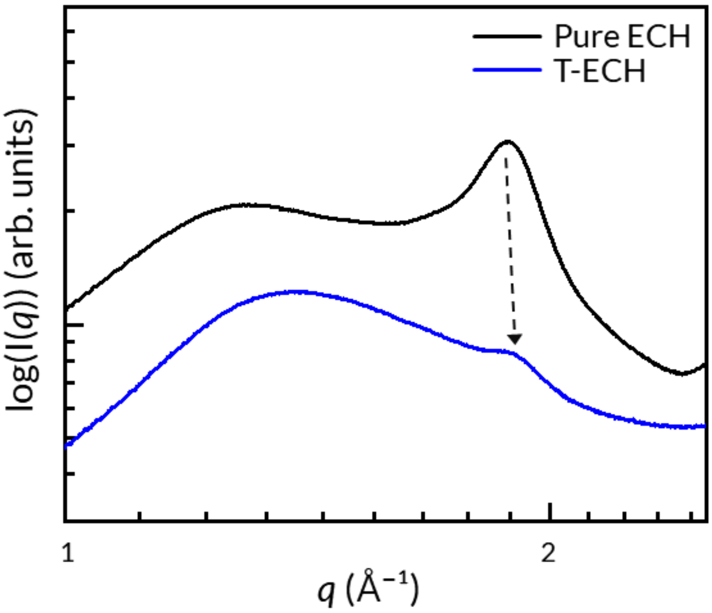

Supplementary Fig. 14| WAXS profile of fully dried Pure ECH and T-ECH.

The peak around q = 1.85 $Å^{-1}$ arises from face-to-face stacking of PEDOT thiophenes[38]. Therefore, the π–π stacking distance of PEDOT can be measured from the q value. The peak q values of Pure ECH and T-ECH are 1.87 and 1.91 $Å^{-1}$, respectively. Therefore, π–π stacking distances of Pure ECH and T-ECH are 3.36 Å and 3.28 Å, respectively ($d = 2^* \pi/q$). The smaller distance between thiophenes in T-ECH indicates that PEDOT-PEDOT interaction is stronger in T-ECH than that of Pure ECH, which results in more stable electrical pathways in the hydrogel state. This phenomenon comes from the confined PEDOT nanofibers in PAA.

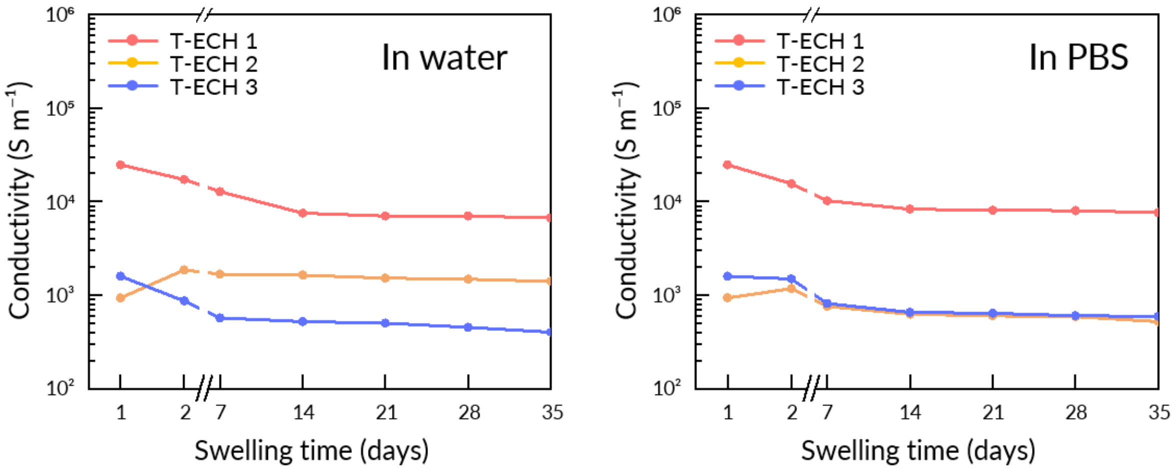

Supplementary Fig. 15| Electrical conductivities in water and PBS with different swelling time. Electrical conductivities of T-ECH 1, 2, and 3 were measured under water and PBS for 35 days.

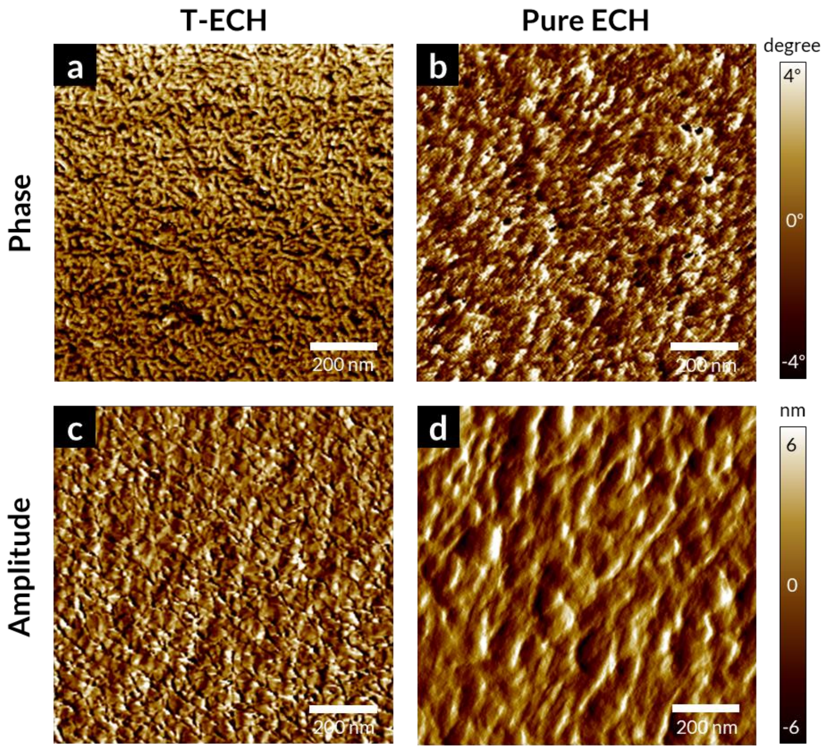

Supplementary Fig. 16| AFM amplitude and phase images of Pure ECH and T-ECH.

Phase images of T-ECH (**a**) and Pure ECH (**b**). Amplitude images of T-ECH (**c**) and Pure ECH (**d**). T-ECH shows a nanofibrous homogeneous network. However, Pure ECH exhibits bulk PEDOT:PSS domains with large aggregates. Scale bar = 200 nm. Similar results were observed in two independent samples.

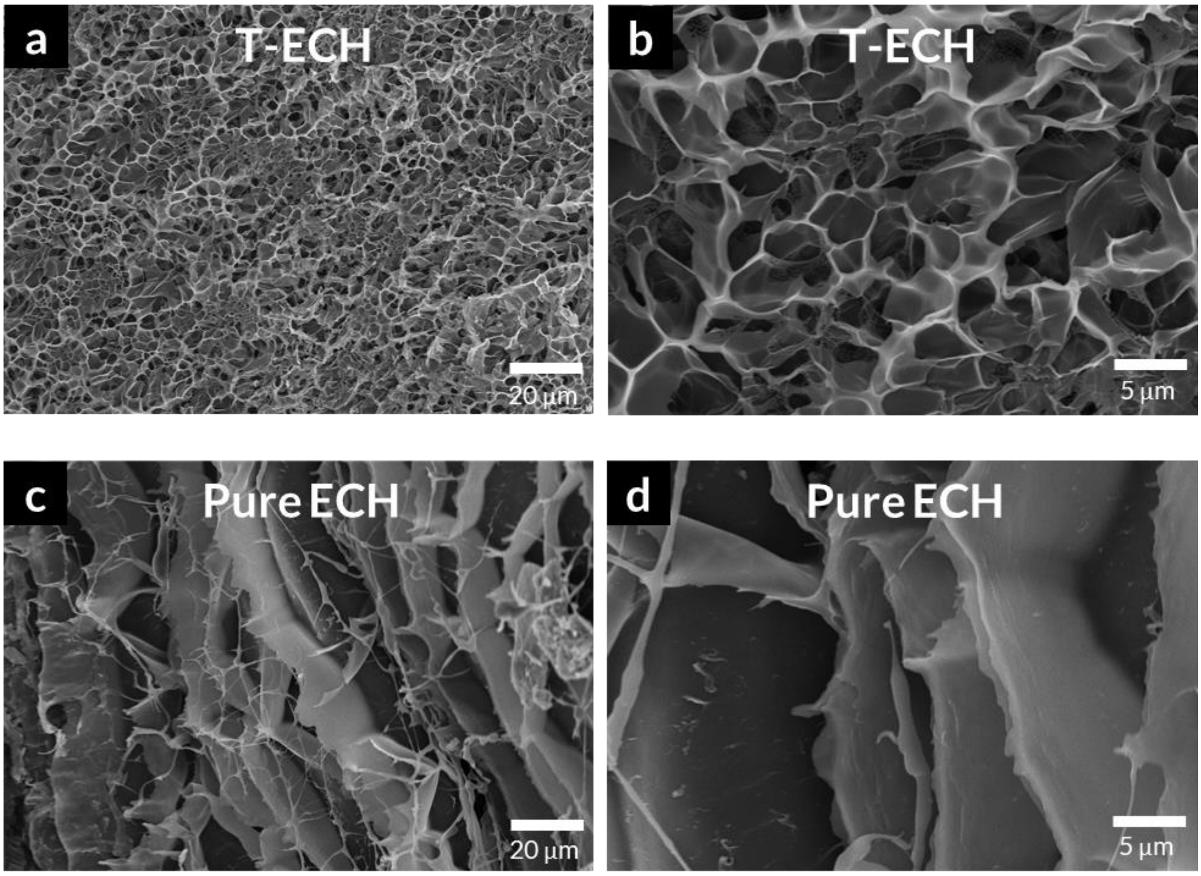

Supplementary Fig. 17| Cross-sectional SEM images of freeze-dried T-ECH and Pure ECH.

a, **b**, SEM images of T-ECH. Homogeneous micro-porous T-ECH has a uniformly interconnected hydrogel structure. **c**, **d**, SEM images of Pure ECH. Pure ECH has a layered structure of bulk, brittle PEDOT:PSS domains. Similar results were observed in two independent experiments.

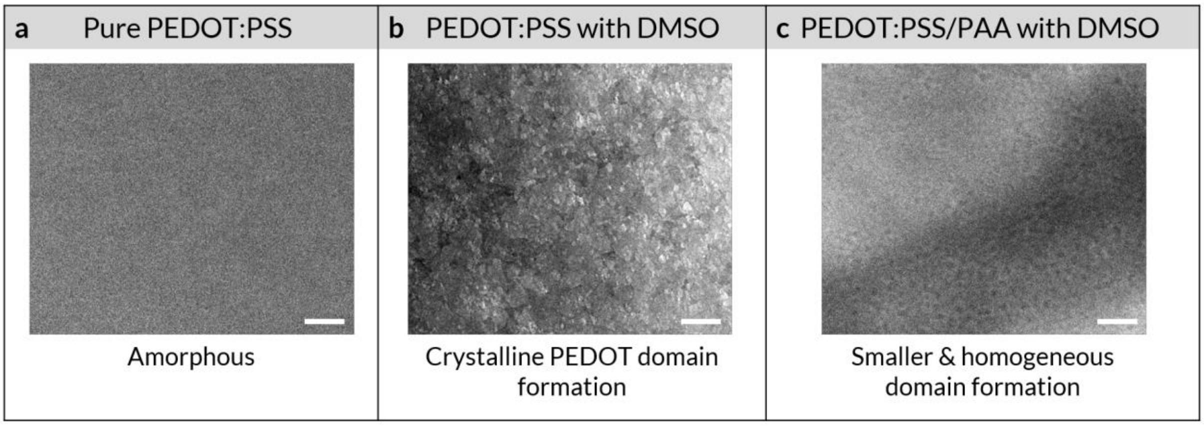

Supplementary Fig. 18| TEM images of pure PEDOT:PSS, PEDOT:PSS with DMSO, and the mixture of PEDOT:PSS, PAA, and DMSO.

a, Pure PEDOT:PSS without any additives shows an amorphous phase. **b**, Then, when DMSO is added, PEDOT:PSS shows crystalline PEDOT domains and phase separation of PEDOT and PSS domains (Pure ECH). **c**, However, the mixture of PAA and PEDOT with DMSO treatment shows a small and homogeneous network due to the template-inducing effect of PAA (T-ECH). Scale bar = 60 nm.

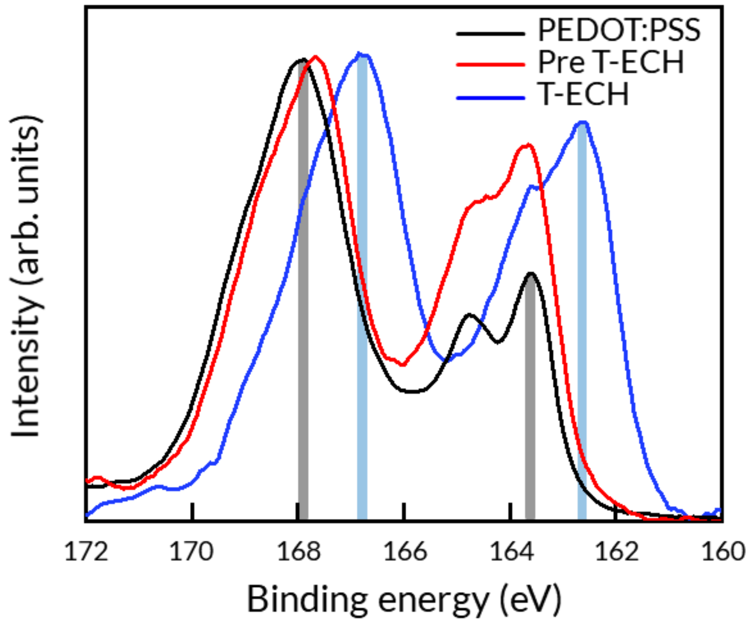

Supplementary Fig. 19| XPS spectra of the fully dried films of Pure ECH, Pre T-ECH, and T-ECH.

In Pre T-ECH, PEDOT:PSS exists in colloidal aggregates without making a fibrous network. However, in T-ECH, PEDOT and PSS form a thin fibrous network along the template network. The thin PEDOT fibers in T-ECH have a more accessible 3-dimensional area than colloidal PEDOT aggregates in Pre T-ECH, resulting in more interaction with polyanions. Therefore, the S 2*p* peak of PEDOT shifts to lower energy. Similarly, PSS can interact more effectively with PAA when they have a structure of a fibrous thin network rather than a bulk aggregate. Therefore, the S 2*p* peak of PSS in T-ECH shifted to lower energy compared to the Pre T-ECH. In addition, the S 2*p* peak of PSS in Pre T-ECH slightly shifted to lower energy than PEDOT:PSS, meaning that PSS forms hydrogen bonding with the PAA template.

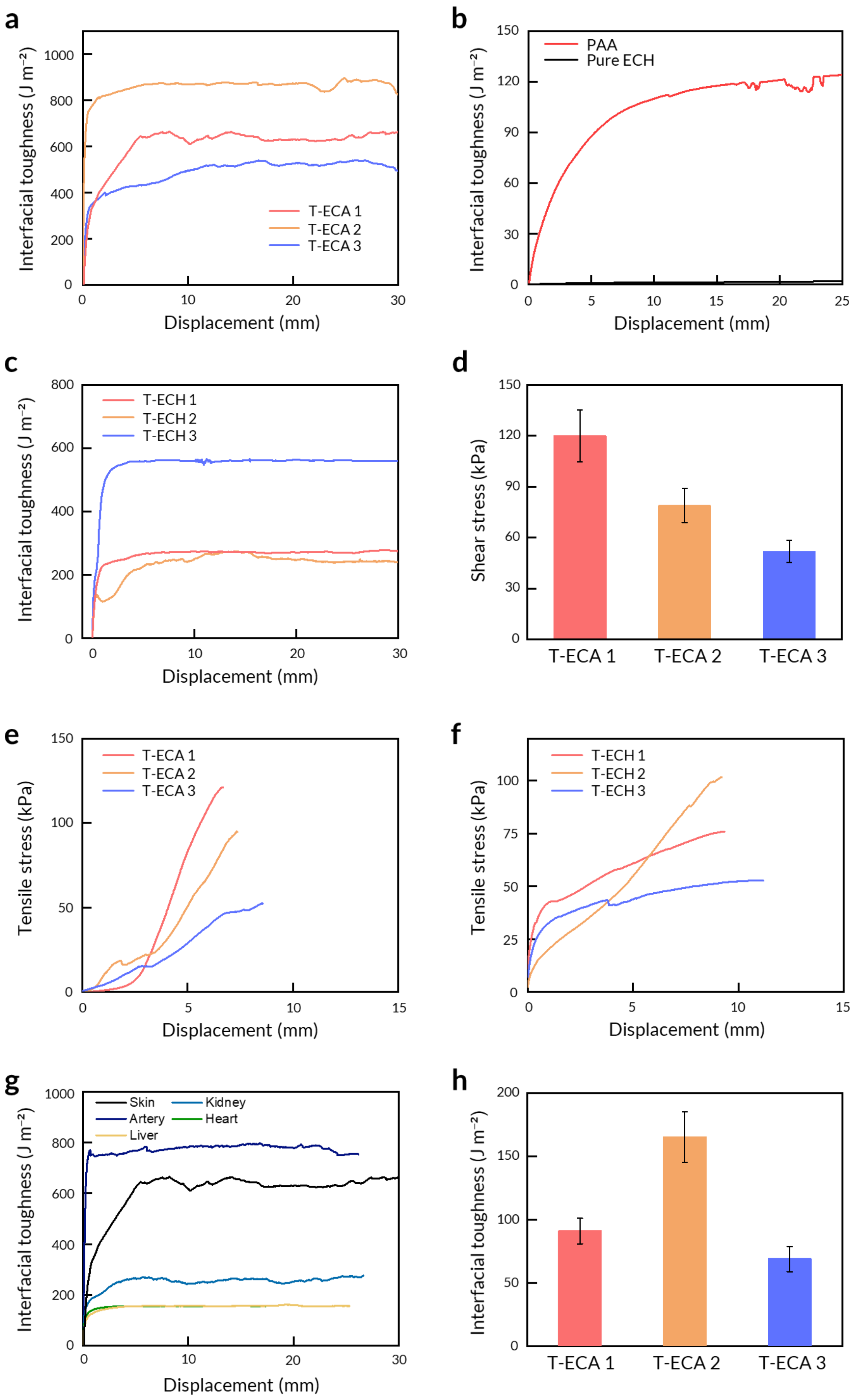
a
Interfacial toughness (J m⁻²)
Displacement (mm)
T-ECA 1
T-ECA 2
T-ECA 3
b
Interfacial toughness (J m⁻²)
Displacement (mm)
PAA
Pure ECH
c
Interfacial toughness (J m⁻²)
Displacement (mm)
T-ECH 1
T-ECH 2
T-ECH 3
d
Shear stress (kPa)
T-ECA 1
T-ECA 2
T-ECA 3
e
Tensile stress (kPa)
Displacement (mm)
T-ECA 1
T-ECA 2
T-ECA 3
f
Tensile stress (kPa)
Displacement (mm)
T-ECH 1
T-ECH 2
T-ECH 3
g
Interfacial toughness (J m⁻²)
Displacement (mm)
Skin
Kidney
Artery
Heart
Liver
h
Interfacial toughness (J m⁻²)
T-ECA 1
T-ECA 2
T-ECA 3

Supplementary Fig. 20| 180 degrees peel test and shear test of T-ECHs and T-ECAs (T-ECH adhesive).

a, Interfacial toughness of T-ECAs with porcine skin measured from a 180 degrees peel test. **b**, Interfacial toughness of PAA and Pure ECH with porcine skin measured from a 180 degrees peel test. **c**, Interfacial toughness of T-ECHs with porcine skin measured from a 180 degrees peel test. **d**, **e**, Shear stress of T-ECAs with porcine skin. Data plotted represents mean and standard deviation (n = 3, n means independent experiments). **f**, Shear stress of T-ECHs with porcine skin. **g**, Interfacial toughness of T-ECAs with various tissues (skin, artery, liver, kidney, and heart) measured from 180 degrees peel test. **h**, Interfacial toughness measured from 180 degrees peel test of T-ECAs with amine-functionalized gold. Data plotted represents mean and standard deviation (n = 3, n means independent experiments).

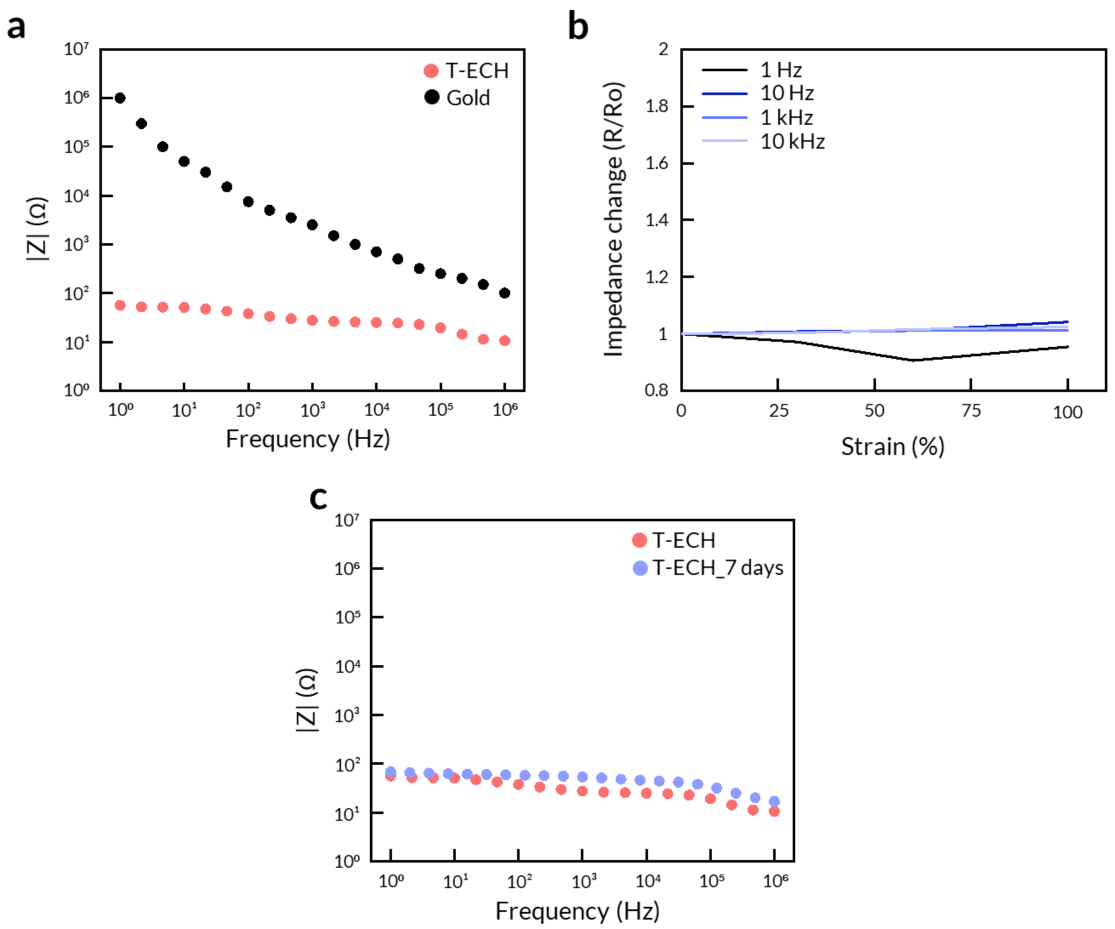

Supplementary Fig. 21| Impedance spectra of T-ECHs.

a, Impedance of T-ECHs. **b**, Impedance change of T-ECH under strain up to 100%. **c**, Impedance of T-ECHs measured after soaking in 7 days in PBS. All tests were done with T-ECH 1.

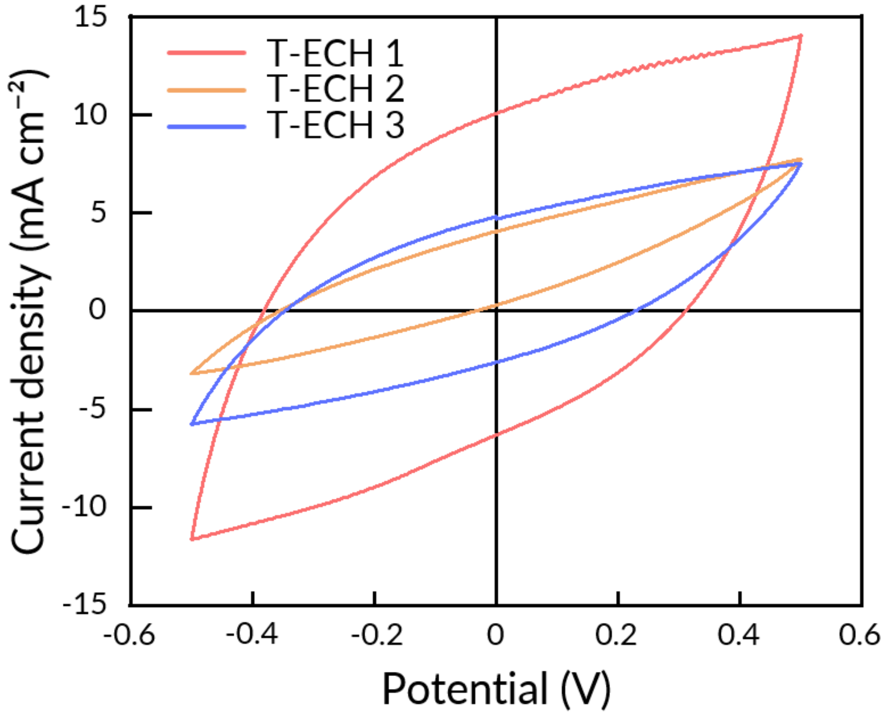

Supplementary Fig. 22| CV of T-ECHs measure in PBS.

CSCs of T-ECH 1, T-ECH 2, and T-ECH 3 calculated from CV are 80 mC/cm², 31 mC/cm², and 39 mC/cm², respectively.

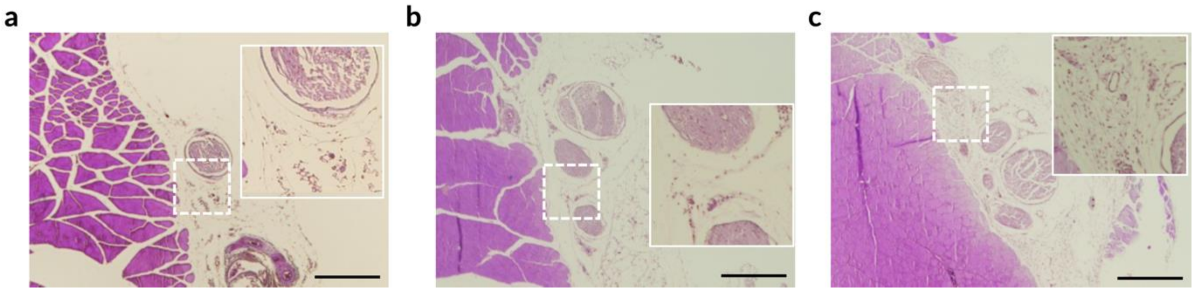

Supplementary Fig. 23| Representative histological images of a, T-ECH electrode **b,** sham group **c,** stainless steel after 2 weeks of implantation (n=3 for each group were examined with similar results). Scale bar = 500 μm. The highest degree of fibrosis was observed in the histology of the stainless steel.

	Acrylic acid : PEDOT:PSS (wt)	Acrylic acid : crosslinker (mol)	Conductivity (S m^{-1})	Modulus (kPa)	Strain at break (%)	Charge storage capacity (mC cm^{-2})
**T-ECH 1**	**10 : 1**	**2,000 : 7**	24765	60	400	79.71
**T-ECH 2**	**20 : 1**	**200 : 1**	934	25	610	31.05
**T-ECH 3**	**20 : 1**	**100 : 1**	1591	30	205	39.09

Supplementary Table 1| Tunable mechanical & electrical properties of T-ECHs.

Mechanical and electrical properties of T-ECH are tunable by changing the amount of crosslinker (MBAA) and PEDOT:PSS content. PEDOT:PSS and crosslinker contents are all compared to the amount of acrylic acid. For example, T-ECH 1 has 0.6 g of acrylic acid and 0.06 g of PEDOT:PSS. T-ECH 2, which has less PEDOT:PSS and crosslinker content of 0.5 mol%, is the softest and most stretchable. With the same amount of PEDOT:PSS as T-ECH 2, T-ECH 3 has higher crosslinker content of 1 mol%. T-ECH 3 exhibits higher electrical conductivity than T-ECH 2 due to the denser template network.

	n value (1/Rg < q < 0.01 Å)	Structure
Pure ECH	3	thick rod; bulk aggregates
PAA	0.76	PAA
T-ECH	1.29	gaussian polymer chain-needle like structure; thin fiber of crystalline PEDOT network formation
PAAm T-ECH	0.85	PAAm; no PEDOT network

Supplementary Table 2| SANS analysis. N values and corresponding structure of hydrogels.

Supplementary References

1. Lu, B. et al., Pure PEDOT:PSS hydrogels. *Nat. Commun.* **10**, 1043 (2019)
2. Feig, V. R., Tran, H., Lee, M. & Bao, Z. Mechanically tunable conductive interpenetrating network hydrogels that mimic the elastic moduli of biological tissue. *Nat. Commun.* **9**, 2740 (2018)
3. Yao, B. et al., Ultrahigh-Conductivity Polymer Hydrogels with Arbitrary Structures. *Adv. Mater.* **29**, 1700974 (2017)
4. Lee, Y. Y. et al. A Strain-Insensitive Stretchable Electronic Conductor: PEDOT:PSS/Acrylamide Organogels. *Adv. Mater.* **28**, 1636-1643 (2016)
5. Zhang, D. et al. Highly stretchable, self-adhesive, biocompatible, conductive hydrogels as fully polymeric strain sensors. *J. Mater. Chem. A* **8**, 20474-20485 (2020)
6. Li, G. et al. Highly Conducting and Stretchable Double-Network Hydrogel for Soft Bioelectronics. *Adv. Mater.* **34**, 2200261 (2022)
7. Suneetha, M. et al. Tissue-adhesive, stretchable, and self-healable hydrogels based on carboxymethyl cellulose-dopamine/PEDOT:PSS via mussel-inspired chemistry for bioelectronic applications. *Chem. Eng. J.* **426**, 130847 (2021)
8. Yu, X. et al. Highly Stretchable, Ultra-Soft, and Fast Self-Healable Conductive Hydrogels Based on Polyaniline Nanoparticles for Sensitive Flexible Sensors. *Adv. Funct. Mater.* **32**, 2204366 (2022)
9. Zhao, Y. S. et al. Hierarchically Structured Stretchable Conductive Hydrogels for High-Performance Wearable Strain Sensors and Supercapacitors. *Matter-Us* **3**, 1196-1210 (2020)
10. Duan, J., Liang, X., Guo, J., Zhu, K. & Zhang, L. Ultra-stretchable and force-sensitive hydrogels reinforced with chitosan microspheres embedded in polymer networks. *Adv. Mater.* **28**, 8037-8044 (2016)
11. Shi, Y., Ma, C., Peng, L. & Yu, G. Conductive “smart” hybrid hydrogels with PNIPAM and nanostructured conductive polymers. Adv. Funct. Mater. 25, 1219-1225 (2015)
12. Chen, J. S., Peng, Q. Y., Thundat, T. & Zeng, H. B. Stretchable, Injectable, and Self-Healing Conductive Hydrogel Enabled by Multiple Hydrogen Bonding toward Wearable Electronics. *Chem. Mater.* **31**, 4553-4563 (2019)
13. Stejskal, J. et al. Polyaniline cryogels supported with poly(vinyl alcohol): soft and conducting. *Macromolecules* **50**, 972-978 (2017)
14. Jin, X. et al. Stretchable, conductive PAni-PAAm-GOCS hydrogels with excellent mechanical strength, strain sensitivity and skin affinity. *Chem. Eng. J.* **394**, 124901 (2020)
15. Zhao, Y. S. et al. Somatosensory actuator based on stretchable conductive photothermally responsive hydrogel. *Sci. Robot.* **6**, eabd5483 (2021)

16. Wang, S. et al. Fabrication of Polypyrrole-Grafted Gelatin-Based Hydrogel with Conductive, Self-Healing, and Injectable Properties. *ACS Appl. Polym. Mater.* **2**, 3016-3023 (2020)

17. Hur, J. et al. Polypyrrole/Agarose-based electronically conductive and reversibly restorable hydrogel. *ACS Nano* **8**, 10066-10076 (2014)

18. Ohm, Y., Pan, C., Ford, M.J. et al. An electrically conductive silver–polyacrylamide–alginate hydrogel composite for soft electronics. *Nat. Electron.* **4**, 185–192 (2021)

19. Jing, X. et al. Stretchable gelatin/silver nanowires composite hydrogels for detecting human motion. *Mater. Lett.* **237**, 53–56 (2019)

20. Feig, V. R., Tran, H., Lee, M. & Bao, Z. Mechanically tunable conductive interpenetrating network hydrogels that mimic the elastic moduli of biological tissue. *Nat. Commun.* **9**, 2740 (2018)

21. Sun, J. Y. et al. Highly stretchable and tough hydrogels. *Nature* **489**, 133–136 (2012)

22. Li, J. Z., Ma, L., Chen, G. X., Zhou, Z. Q. & Li, F. A high water-content and high elastic dual-responsive polyurethane hydrogel for drug delivery. *J. Mater. Chem. B* **3**, 8401-8409 (2015)

23. Ye, T. T. et al. A Tissue-Like Soft All-Hydrogel Battery. *Adv. Mater.* **34**, 2105120 (2021)

24. Liu, C. et al. Tough hydrogels with rapid self-reinforcement. *Science* **372**, 1078–1081 (2021)

25. Lin, S. T., Liu, J., Liu, X. Y. & Zhao, X. H. Muscle-like fatigue-resistant hydrogels by mechanical training. *Proc. Natl. Acad. Sci. U.S.A.* **116**, 10244-10249 (2019)

26. Sun, T. L. et al. Physical hydrogels composed of polyampholytes demonstrate high toughness and viscoelasticity. *Nat. Mater.* **12**, 932–937 (2013)

27. Wang, S. H. et al. Strong, tough, ionic conductive, and freezing-tolerant all-natural hydrogel enabled by cellulose-bentonite coordination interactions. *Nat. Commun.* **13**, 3408 (2022)

28. Means, A. K., Shrode, C. S., Whitney, L. V., Ehrhardt, D. A. & Grunlan, M. A. Double Network Hydrogels that Mimic the Modulus, Strength, and Lubricity of Cartilage. *Biomacromolecules* **20**, 2034–2042 (2019)

29. Hua, M. T. et al. Strong tough hydrogels via the synergy of freeze-casting and salting out. *Nature* **590**, 594–599 (2021)

30. Lin, S. et al. Anti-fatigue-fracture hydrogels. *Sci. Adv.* **5**, eaau8528 (2019)

31. Ji, D. et al. Superstrong, superstiff, and conductive alginate hydrogels. *Nat. Commun.* **13**, 3019 (2022)

32. Rauner, N., Meuris, M., Zoric, M. & Tiller, J. C. Enzymatic mineralization generates ultrastiff and tough hydrogels with tunable mechanics. *Nature* **543**, 407–410 (2017)

33. Zhang, X. N. et al. A Tough and Stiff Hydrogel with Tunable Water Content and Mechanical Properties Based on the Synergistic Effect of Hydrogen Bonding and Hydrophobic Interaction. *Macromolecules* **51**, 8136-8146 (2018)

34. Zhang, G., Kim, J., Hassan, S. & Suo, Z. Self-assembled nanocomposites of high water content and load-bearing capacity. *Proc. Natl. Acad. Sci. U.S.A.* **119**, e2203962119 (2022)

35. Li, J. et al. Tough adhesives for diverse wet surfaces. *Science* **357**, 378–381 (2017)

36. Yan, X. et al. Approaching disorder-tolerant semiconducting polymers. *Nat. Commun.* **12**, 5723 (2021)

37. Patel, S. N. et al. Morphology controls the thermoelectric power factor of a doped semiconducting polymer. *Sci. Adv.* **3**, e1700434 (2017)

38. Massonnet, N., Carella, A., de Geyer, A., Faure-Vincent, J. & Simonato, J. P. Metallic behaviour of acid doped highly conductive polymers. *Chem. Sci.* **6**, 412-417 (2015)
