# Supplementary material for: Highly conductive tissue-like hydrogel interface through template-directed assembly
Source: Nat Commun. 2023 Apr 18;14:2206. doi: 10.1038/s41467-023-37948-1 (PMC10113367; doi:10.1038/s41467-023-37948-1)
Supplement: Supplementary file 3 — Description of Additional Supplementary Files [file 41467_2023_37948_MOESM3_ESM.pdf]

Description of Additional Supplementary Files

File Name: Supplementary Movie 1
Description: Compression test of T-ECH

File Name: Supplementary Movie 2
Description: Nerve stimulation using T-ECH electrodes.
