# Supplementary material for: Highly conductive tissue-like hydrogel interface through template-directed assembly
Source: Nat Commun. 2023 Apr 18;14:2206. doi: 10.1038/s41467-023-37948-1 (PMC10113367; doi:10.1038/s41467-023-37948-1)
Supplement: Supplementary file 6 — Reporting Summary [file 41467_2023_37948_MOESM6_ESM.pdf]

nature portfolio

Corresponding author(s): Jiheong Kang

Last updated by author(s): Feb 22, 2023

Reporting Summary

Nature Portfolio wishes to improve the reproducibility of the work that we publish. This form provides structure for consistency and transparency in reporting. For further information on Nature Portfolio policies, see our Editorial Policies and the Editorial Policy Checklist.

Statistics

For all statistical analyses, confirm that the following items are present in the figure legend, table legend, main text, or Methods section.

n/a	Confirmed	
☐	☒	The exact sample size (*n*) for each experimental group/condition, given as a discrete number and unit of measurement
☐	☒	A statement on whether measurements were taken from distinct samples or whether the same sample was measured repeatedly
☐	☒	The statistical test(s) used AND whether they are one- or two-sided<br>*Only common tests should be described solely by name; describe more complex techniques in the Methods section.*
☐	☒	A description of all covariates tested
☐	☒	A description of any assumptions or corrections, such as tests of normality and adjustment for multiple comparisons
☐	☒	A full description of the statistical parameters including central tendency (e.g. means) or other basic estimates (e.g. regression coefficient) AND variation (e.g. standard deviation) or associated estimates of uncertainty (e.g. confidence intervals)
☐	☒	For null hypothesis testing, the test statistic (e.g. *F*, *t*, *r*) with confidence intervals, effect sizes, degrees of freedom and *P* value noted<br>*Give P values as exact values whenever suitable.*
☒	☐	For Bayesian analysis, information on the choice of priors and Markov chain Monte Carlo settings
☒	☐	For hierarchical and complex designs, identification of the appropriate level for tests and full reporting of outcomes
☒	☐	Estimates of effect sizes (e.g. Cohen's *d*, Pearson's *r*), indicating how they were calculated

Our web collection on statistics for biologists contains articles on many of the points above.

Software and code

Policy information about availability of computer code

Data collection	NIS Elements AR (Nikon) was used to collect the immunohistochemistry images.
Data analysis	GraphPad Prism 9 software was used to analyze statistical significance for all comparison studies.<br>FIJI (ImageJ; version 1.0) was used to quantify the intensity of fluorescence for immunohistochemistry analysis.

For manuscripts utilizing custom algorithms or software that are central to the research but not yet described in published literature, software must be made available to editors and reviewers. We strongly encourage code deposition in a community repository (e.g. GitHub). See the Nature Portfolio guidelines for submitting code & software for further information.

Data

Policy information about availability of data

All manuscripts must include a data availability statement. This statement should provide the following information, where applicable:

- Accession codes, unique identifiers, or web links for publicly available datasets
- A description of any restrictions on data availability
- For clinical datasets or third party data, please ensure that the statement adheres to our policy

The data that support the findings of this study are available within this article and its Supplementary Information. Source data is provided as Source Data file. Data is also available from the corresponding author upon request.

Human research participants

Policy information about studies involving human research participants and Sex and Gender in Research.

Reporting on sex and gender	n/a
Population characteristics	n/a
Recruitment	n/a
Ethics oversight	n/a

Note that full information on the approval of the study protocol must also be provided in the manuscript.

Field-specific reporting

Please select the one below that is the best fit for your research. If you are not sure, read the appropriate sections before making your selection.

[x] Life sciences [ ] Behavioural & social sciences [ ] Ecological, evolutionary & environmental sciences

For a reference copy of the document with all sections, see nature.com/documents/nr-reporting-summary-flat.pdf

Life sciences study design

All studies must disclose on these points even when the disclosure is negative.

Sample size	Sample size n=9 for sciatic nerve stimulation was determined following the previously reported neural stimulation interface studies (Ref 7 and 45. of manuscript).
Data exclusions	No data was excluded from the analysis.
Replication	The reproducibility of the experiments were confirmed with similar observations by the independent experiments and iterations with representatives.
Randomization	All experiments were performed with randomly allocated experimental groups.
Blinding	All experiments and data collection was conducted as blinded including group allocation. All analyses were blinded to the experimental conditions.

Reporting for specific materials, systems and methods

We require information from authors about some types of materials, experimental systems and methods used in many studies. Here, indicate whether each material, system or method listed is relevant to your study. If you are not sure if a list item applies to your research, read the appropriate section before selecting a response.

Materials & experimental systems

n/a	Involved in the study	
[ ]	[x]	Antibodies
[x]	[ ]	Eukaryotic cell lines
[x]	[ ]	Palaeontology and archaeology
[ ]	[x]	Animals and other organisms
[x]	[ ]	Clinical data
[x]	[ ]	Dual use research of concern

Methods

n/a	Involved in the study	
[x]	[ ]	ChIP-seq
[x]	[ ]	Flow cytometry
[x]	[ ]	MRI-based neuroimaging

Antibodies

Antibodies used	Anti-S100 beta antibody (abcam, ab52642), Anti-160kD Neurofilament Medium antibody (abcam, ab195658), Anti-Iba1 antibody (abcam, ab107159), Recombinant Anti-TNF alpha antibody (abcam, ab215188), Donkey Anti-Rabbit IgG H&L, Alexa Flour 594 (abcam, ab150076), Donkey Anti-Goat IgG H&L, Alexa Flour 594 (abcam, ab150129)

Validation	All antibodies used in the study are commonly utilized. Manufacturer suggests that primary antibodies (ab52642, ab1956658, ab107159) react with the mouse and suitable for the immunohistochemistry.

Animals and other research organisms

Policy information about studies involving animals; ARRIVE guidelines recommended for reporting animal research, and Sex and Gender in Research

Laboratory animals	Male C57BL/6 mice aged 8-10 weeks and female SD rats aged 10-12 weeks (Koatech, South Korea) were used for the study. Mice and rats were housed and maintained under conditions of 12h light/dark cycle and 22-24 °C, and given ad libitum access to food and water.
Wild animals	The study did not involve wild animals.
Reporting on sex	The study did not involve sex-based analysis.
Field-collected samples	The study did not involve samples collected from the field.
Ethics oversight	All animal procedures were guided and approved by Institutional Animal Care and Use Committee (IACUC) of KAIST.

Note that full information on the approval of the study protocol must also be provided in the manuscript.
